# Heavy Metal Contamination in Commercial Coffee Products in Saudi Arabia: Prevalence, Carcinogenic and Non-Carcinogenic Risk Assessment, and Implications for Diabetes and Cardiovascular Disease

**DOI:** 10.3390/toxics14090761

**Published:** 2026-08-26

**Authors:** Azizah A. Algreiby, Suzan Makawi, Ahmed H. Bakheit, Abdullah H. Alluhayb

**Affiliations:** 1 Department of Chemistry, College of Science, Qassim University, Buraidah 51452, Saudi Arabia; grieby@qu.edu.sa (A.A.A.); s.alkhaleefa@qu.edu.sa (S.M.); 2 Department of Pharmaceutical Chemistry, College of Pharmacy, King Saud University, Riyadh 11451, Saudi Arabia; abakheit@ksu.edu.sa; 3 Department of Chemistry, Faculty of Science and Technology, Al-Neelain University, P.O. Box 1270, Khartoum 12702, Sudan

**Keywords:** heavy metals, coffee, Saudi Arabia, diabetes mellitus, carcinogenic risk, cadmium, lead, arsenic, cardiovascular disease, ICP-M

## Abstract

Coffee is deeply embedded in Saudi culture and is among the most consumed beverages worldwide. However, contamination by toxic heavy metals may represent an underrecognized public health concern, particularly in regions already burdened by diabetes mellitus and cardiovascular disease. This study evaluated concentrations of lead (Pb), cadmium (Cd), arsenic (As), chromium (Cr), and nickel (Ni) in 21 commercial coffee samples representing seven product categories marketed in Saudi Arabia. Samples were prepared using EPA Method 3050B acid digestion and analyzed by inductively coupled plasma mass spectrometry (ICP-MS). Health risks were assessed through estimated daily intake (EDI), hazard quotient (HQ), hazard index (HI), and incremental lifetime cancer risk (ILCR) using USEPA exposure parameters and Saudi-specific coffee consumption data. All metals were detected in every sample. Mean concentrations (mg/kg dry weight) were 1.638 for Pb, 0.560 for Cd, 0.622 for Cr, 0.536 for Ni, and 0.034 for As. Cadmium exceeded the European Union maximum limit for roasted coffee in all samples, while elevated Pb levels were observed in dark roast and Yemeni coffees. Although the overall non-carcinogenic risk remained below the accepted threshold (HI = 0.438), the combined ILCR for As, Cd, and Pb was 5.84 × 10^−5^. Inclusion of a conservative screening-level chromium contribution, calculated by assuming that all measured total Cr was present as Cr(VI), increased the upper-bound total ILCR to 9.38 × 10^−5^. This value remained within, but approached the upper boundary of, the risk-management range applied in this study. These findings highlight the need for strengthened monitoring and regulatory control of coffee products in Saudi Arabia.

## 1. Introduction

Coffee (*Coffea arabica* L. and *Coffea canephora* Pierre ex Froehner) is the second most traded commodity globally after petroleum, with annual production exceeding 175 million 60 kg bags worldwide. Asia consumed approximately 2794 kt of coffee in 2023 (FAOSTAT data), reflecting a region-wide surge in consumption. Within this broader landscape, Saudi Arabia has emerged as a particularly significant market. According to FAOSTAT-based data, Saudi Arabia reached a per capita consumption of 5.39 kg/person in 2023, ranking 67th globally, a figure that substantially exceeds neighboring countries such as the UAE (0.640 kg/person) and approaches Bahrain (6.66 kg/person) [1].

The Saudi coffee market was estimated at approximately SAR 4.1 billion (USD ~1.09 billion) in 2024, with projected annual growth of 4.37%, anticipated to reach SAR 5.3 billion by 2030. Average individual annual expenditure on coffee is projected to reach SAR 420 by 2030. This remarkable market expansion is driven by population growth, rising consumer spending, proliferation of specialty cafés, robust e-commerce platforms, and growing demand for both instant and single-origin roasted coffee. Local varieties, particularly Khawlani coffee from the Jazan region, add a culturally significant domestic production dimension [2].

Arabic coffee is a culturally important beverage in Saudi Arabia and is commonly served in homes and social gatherings, often with additives such as cardamom, milk, cloves, or saffron and accompanied by foods such as dates and chocolate. Although coffee contains bioactive compounds, including caffeine, antioxidants, and anti-inflammatory constituents, its health effects may vary according to preparation method, frequency of intake, additives, and accompanying dietary habits. Evidence from the Eastern Province of Saudi Arabia indicates that Arabic coffee is widely consumed, with more than half of participants reporting daily intake, and that obesity and overweight were common among consumers. The study found significant associations between BMI and coffee-drinking frequency, number of cups consumed, use of additives such as milk and cardamom, and eating chocolate with coffee, suggesting that the broader pattern of Arabic coffee consumption may contribute to excess caloric intake and obesity risk rather than coffee alone. These findings highlight the need to examine Arabic coffee consumption within the Saudi dietary context, particularly when evaluating obesity and related cardiometabolic risk factors [3].

Of particular cultural relevance is Arabic coffee (qahwa), which is commonly prepared from lightly roasted coffee beans, frequently with cardamom, and served during social and hospitality occasions in Saudi Arabia. The present study quantified metals in dry coffee products before brewing, reheating, or contact with preparation and serving vessels. Accordingly, the reported concentrations represent contamination already present in the dry coffee material and do not account for changes that may occur during beverage preparation. The metal concentration in brewed qahwa could differ from that measured in the dry material because only a fraction of each metal may be extracted from the coffee during brewing, while additional contributions could potentially arise from water [4], additives, grinding equipment, or contact with traditional metal vessels known as dallah [5]. Previous research has shown that some cookware materials can release metals during heating and that migration may be influenced by vessel composition, age, surface damage, repeated use, and contact time [5]. However, the present study did not analyze brewed qahwa or assess metal migration from dallah vessels. Consequently, dallah use should be regarded as a plausible but unconfirmed additional source of metal exposure, and the actual metal concentrations in the consumed beverage require direct experimental determination [2,6].

Aluminum exposure has also attracted attention because of its neurotoxic potential and its proposed association with neurodegenerative disorders, particularly Alzheimer’s disease. Experimental evidence suggests that aluminum may promote several biological processes relevant to Alzheimer’s disease, including oxidative stress, mitochondrial dysfunction, neuroinflammation, amyloid-β aggregation, impaired amyloid clearance, and tau phosphorylation. However, epidemiological studies have produced inconsistent results, and a direct causal relationship between aluminum exposure and Alzheimer’s disease has not been established. Aluminum should therefore be considered a possible contributing environmental factor within the multifactorial pathogenesis of Alzheimer’s disease rather than a confirmed independent cause [7,8,9].

Diabetes is a major and rapidly expanding global public health burden. According to the Global Burden of Disease Study 2021, an estimated 529 million people were living with diabetes worldwide in 2021, with a global age-standardized prevalence of 6.1%; this burden is projected to more than double to 1.31 billion people by 2050 [10,11]. The increase is driven predominantly by type 2 diabetes, which accounted for approximately 96% of all diabetes cases and 95.4% of diabetes-related DALYs in 2021, with a high body mass index identified as the leading attributable risk factor, responsible for 52.2% of global type 2 diabetes DALYs. The burden is particularly high in North Africa and the Middle East, where age-standardized diabetes prevalence reached 9.3% in 2021 and is projected to rise to 16.8% by 2050; in Saudi Arabia, prevalence was estimated at 11.3% in 2021, with diabetes DALYs increasing substantially between 1990 and 2021. These findings highlight the urgent need for prevention-focused strategies targeting obesity, unhealthy diets, physical inactivity, early diagnosis, and improved diabetes care globally and in Saudi Arabia [12]. The disease burden is distributed unevenly across income strata: prevalence is highest in middle-income countries, followed by high-income and then low-income nations. A pooled analysis of 100 countries revealed stark disparities in care access: diabetes diagnosis in 2021 ranged from 35.3% in low-income countries to 69.9% in high-income countries, while statin use ranged from 9.7% to 58.7% across the same income spectrum [13].

Diabetes mellitus constitutes one of the most pressing non-communicable diseases (NCD) crises of the 21st century. The International Diabetes Federation (IDF) estimates that 589 million adults aged 20–79 were living with diabetes in 2024, a figure projected to escalate dramatically to 853 million by 2050, representing a global adult prevalence of approximately 11.1% [11,14]. The economic burden is staggering: in 2024, global health expenditure attributable to diabetes exceeded USD 966 billion.

The disease burden is distributed unevenly across income strata: prevalence is highest in middle-income countries, followed by high-income and then low-income nations. A pooled analysis of 100 countries revealed stark disparities in care access: diabetes diagnosis in 2021 ranged from 35.3% in low-income countries to 69.9% in high-income countries, while statin use ranged from 9.7% to 58.7% across the same income spectrum.

The MENA region presents a particularly alarming picture: 1 in 6 adults in the region has diabetes, accounting for approximately 85 million affected individuals and representing the highest regional diabetes prevalence in the world at 17.6% [11]. Within the Gulf Cooperation Council (GCC), estimated diabetes-related deaths among adults aged 20–79 in 2024 were: Saudi Arabia ~8038 deaths (diabetes accounting for 14.5% of deaths in this age group), Kuwait ~924 (14.8%), Qatar ~364 (15.0%), Bahrain ~338 (13.7%), Oman ~625 (10.1%), and UAE ~830 (10.9%). Several Gulf countries also rank among the world’s highest childhood type 1 diabetes incidence rates globally, reflecting both genetic susceptibility and environmental contributors.

Beyond diabetes alone, the WHO estimates that NCDs killed at least 43 million people in 2021, approximately 75% of all non-pandemic-related deaths worldwide; some 18 million of these were premature deaths before age 70, with 82% occurring in low- and middle-income countries. This context underscores the urgency of identifying modifiable environmental contributors to diabetes and cardiovascular disease risk, including dietary exposures to toxic heavy metals through commonly consumed foods and beverages.

Heavy metals including lead (Pb), cadmium (Cd), arsenic (As), mercury (Hg), chromium (Cr), and nickel (Ni) enter the food chain through multiple routes: anthropogenic soil contamination from mining, industrial effluents and atmospheric deposition; naturally occurring geological enrichment in volcanic or metalliferous soils; agricultural inputs including phosphate fertilizers with Cd impurities; and leaching from metal cooking, storage, and brewing vessels. Coffee plants (*Coffea* spp.) are particularly susceptible to soil bioaccumulation of heavy metals, as they preferentially uptake cadmium and other divalent cations via root transporters shared with essential micronutrients such as zinc and iron [15].

Contaminated foods and beverages, including coffee containing heavy metals such as lead, cadmium, mercury, and arsenic, may contribute to higher risks of both diabetes mellitus and arterial/cardiovascular diseases through several well characterized biological mechanisms. These include: (i) generation of reactive oxygen species (ROS) and depletion of antioxidant defenses (glutathione, superoxide dismutase, catalase), leading to oxidative damage in pancreatic islets and vascular endothelium; (ii) direct toxic effects on pancreatic β-cells, inducing apoptosis, degranulation, and impaired insulin synthesis and secretion; (iii) interference with insulin signaling pathways, including PI3K/Akt phosphorylation, GLUT4 translocation, and peripheral glucose uptake; (iv) displacement of essential metal cofactors (particularly cadmium competing with zinc in insulin crystallization and secretion); (v) activation of pro-inflammatory signaling pathways (NF-κB, IL-6, TNF-α) that promote insulin resistance; and (vi) endothelial dysfunction, LDL oxidation, and acceleration of atherosclerosis relevant to cardiovascular disease [16,17,18].

A cross-sectional analysis of NHANES 2013–2016 data found that blood or serum levels of lead, cadmium, and copper were significantly higher in adults with diabetes compared with healthy controls, and that exposure to metal mixtures was positively associated with both prevalent diabetes and elevated HbA1c levels. Prospective evidence from the Study of Women’s Health Across the Nation (SWAN) demonstrated that each doubling of urinary arsenic concentration was associated with a 19% increased diabetes risk (HR = 1.19, 95% CI: 1.10–1.30), and each doubling of urinary lead was associated with a 20% increased risk (HR = 1.20, 95% CI: 1.05–1.37) over 16 years of follow-up. A meta-analysis of 22 studies (*N* = 55,536) confirmed that mercury showed the strongest pooled effect size for metabolic syndrome association (ES = 1.26, 95% CI: 1.06–1.48) [19].

The heavy metal content of brewed coffee is determined by an interplay of three major factors: (i) the concentration of metals in the green bean, which reflects the bioavailability of metals from soil in the country of origin; (ii) transformations during the roasting process; and (iii) leaching from brewing equipment during preparation [20].

Roasting degrees modulates the contamination and toxicity profile in several distinct ways. First, as coffee undergoes progressively darker roasting, significant mass is lost (15–20% in light roast; up to 25–30% in dark roast) through evaporation of water and organic matter. Since non-volatile heavy metals are retained during roasting while water and volatile organic matter are lost, their concentration per unit mass of roasted coffee may increase relative to that in the corresponding green beans, particularly at darker roasting levels. Second, acrylamide (formed via the Maillard reaction) is present at higher concentrations in light roasts and decreases with increasing roast temperature; conversely, furan and other thermal degradation products are more prevalent in darker roasts. Third, and critically for antioxidant capacity, the chlorogenic acids that constitute the primary bioactive protective compounds in coffee associated with improved insulin sensitivity, β-cell protection, and anti-inflammatory effects are progressively degraded by heat during dark roasting, with losses exceeding 70% in espresso-level roasts compared to light roasts. This means dark roasts may simultaneously carry higher heavy metal concentrations per unit mass and lower concentrations of the protective antioxidant compounds that otherwise partially mitigate health risks from moderate coffee consumption [20].

Brewing vessel composition further compounds contamination risk. Traditional copper and brass dallah pots used for qahwa preparation, improperly tinned interior surfaces, corroded aluminum vessels, and low-grade stainless steel with inadequate nickel-chromium alloying can all leach metal ions into the hot, acidic coffee brew. The acidic pH of coffee (typically 4.5–5.5) acts as an efficient extractant for metal ions from reactive metallic surfaces. Prolonged contact times during traditional slow brewing and repeated reheating of the same preparation, both common practices in qahwa culture, amplify metal leaching substantially. Aluminum leaching in particular has been documented from aluminum moka pots and traditional brewing vessels when used with acidic coffee brews, and aluminum toxicity has been proposed as a contributor to insulin resistance through interference with insulin receptor signaling [21].

Despite the substantial and growing coffee consumption in Saudi Arabia, the public health implications of heavy metal contamination in commercially available coffee products remain poorly characterized in the Kingdom. Given the alarming diabetes and cardiovascular disease burden in the GCC, there is an urgent need for data-driven risk assessment specific to this high-consumption population. The present study therefore aimed to: (1) quantify concentrations of Pb, Cd, As, Cr, and Ni in seven categories of commercial coffee products available in the Saudi market using ICP-MS; (2) compare detected concentrations with international maximum limits (EU Regulation 1881/2006 and amendments; Codex Alimentarius; JECFA provisional tolerable weekly intakes); (3) calculate estimated daily intakes (EDI), hazard quotients (HQ), hazard index (HI), and incremental lifetime cancer risks (ILCR) using USEPA methodologies calibrated to Saudi Arabia’s per capita coffee consumption; and (4) provide a comprehensive mechanistic discussion of how the observed contamination levels may contribute to the elevated risks of type 2 diabetes and cardiovascular disease in the Saudi population.

## 2. Materials and Methods

### 2.1. Sample Collection

Twenty-one commercial coffee samples were collected from retail supermarkets, specialty coffee shops, and local markets across Saudi Arabia between 2024 and 2025. Seven distinct product categories were represented, with three replicate packages/batches per category to account for inter-batch variability: (i) Brazilian-origin roasted coffee (Br, *n* = 3); (ii) Ethiopian-origin roasted coffee (ET, *n* = 3); (iii) Saudi-packaged ground coffee (S-P, *n* = 3); (iv) blended ground coffee of mixed origin (B-P, *n* = 3); (v) dark-roast coffee (D-P, *n* = 3); (vi) Yemeni-origin coffee (YE, *n* = 3); and (vii) Arabic/cardamom coffee (qahwa; CA-P, *n* = 3). All samples were purchased in sealed, commercially packaged form. Samples were stored in their original sealed packaging at room temperature (20–22 °C) in the dark until analysis. Packaging details, origin countries, and batch numbers were recorded.

### 2.2. Sample Digestion

Sample preparation followed United States Environmental Protection Agency Method 3050B (Acid Digestion of Sediments, Sludges, and Soils, Revision 2; USEPA, 2007), adapted for food matrices. Approximately 1.000 g (±0.001 g) of each homogenized coffee sample was accurately weighed into a clean Pyrex^®^ digestion flask. Ten milliliters of concentrated analytical-grade nitric acid (HNO_3_, ≥69%; Sigma-Aldrich, St. Louis, MO, USA) were added, and the mixture was heated gently on a calibrated hot plate at approximately 80 °C for 15 min to allow initial dissolution of organic matter, before the temperature was gradually raised to 120 °C.

Once vigorous reaction subsided, 3 mL of 30% hydrogen peroxide (H_2_O_2_; Sigma-Aldrich, USA) were added dropwise to complete the oxidative digestion. Heating continued at 120 °C until a clear or pale-yellow solution was obtained, indicating complete mineralization of organic carbon. The digest was then allowed to cool, quantitatively transferred to a 50 mL volumetric flask, and made up to volume with ultrapure deionized water (resistivity ≥ 18.2 MΩ·cm; ultrapure water obtained using a Milli-Q^®^ EQ 7000 system (Merck Millipore, Darmstadt, Germany)). The solution was filtered through a 0.45 μm PTFE membrane filter prior to analysis. Procedural blanks containing HNO_3_ and H_2_O_2_ without a coffee sample were processed in parallel with each analytical batch to monitor potential contamination introduced during digestion and handling. An ICP multi-element certified reference standard solution (TraceCERT^®^; Sigma-Aldrich, USA) containing Cr, Ni, As, Cd, and Pb was used to prepare the working calibration standards and to fortify selected coffee samples for pre-digestion matrix-spike recovery experiments. Working calibration solutions were prepared daily by appropriate dilution with ultrapure water and acid matching.

### 2.3. Instrumental Analysis by ICP-MS

Heavy metal concentrations were determined using an Agilent 7900 inductively coupled plasma mass spectrometer (ICP-MS; Agilent 7900 ICP-MS (Agilent Technologies, Santa Clara, CA, USA)) at the Central Laboratory, Department of Chemistry, College of Science, Qassim University, Saudi Arabia. The instrument was equipped with a MicroMist nebulizer and a peristaltic pump for sample introduction. The operating conditions were as follows: radiofrequency generator power, 1550 W; plasma gas flow rate, 15 L/min; carrier gas flow rate, 5 L/min; make-up gas flow rate, 0.15 L/min; nebulizer pump speed, 0.1 rps; three points per peak; three replicate measurements; and an integration time of 0.3 s per mass. High-purity argon (99.9999%; White Martins, Saudi Arabia) was used as the plasma, nebulizer, and auxiliary gas.

The monitored isotopes were ^52^Cr, ^60^Ni, ^75^As, ^111^Cd, and ^208^Pb. The lead isotopes ^206^Pb and ^207^Pb were also monitored to confirm isotopic consistency and assess potential spectral interference. Before analysis, the ICP-MS was tuned and optimized using an appropriate ICP-MS tuning solution. External calibration was performed using working standard solutions at concentrations of 0, 1, 3, 5, 10, 50, 100, and 150 µg/L, prepared by appropriate dilution of the ICP multi-element certified reference standard solution (TraceCERT^®^; Sigma-Aldrich, USA). Calibration linearity was considered acceptable when the coefficient of determination was R^2^ > 0.999. Calibration standards, procedural blanks, quality-control solutions, and sample results were reviewed before quantification. Metal concentrations were corrected for procedural blank contributions and expressed as mg/kg dry weight (d.w.).

### 2.4. Quality Assurance and Quality Control

Analytical quality-control procedures included external calibration, procedural blanks, and replicate instrumental measurements. Working calibration solutions were prepared from a certified ICP multi-element standard solution containing Cr, Ni, As, Cd, and Pb. Calibration linearity was evaluated over the concentration range used for quantification, and a coefficient of determination of R^2^ > 0.999 was considered acceptable. Procedural blanks containing the digestion reagents without a coffee sample were processed with each analytical batch to monitor contamination introduced during sample preparation and analysis. Sample concentrations were corrected for the corresponding procedural blank contributions. Each digest was measured in replicate by ICP-MS, and analytical precision was expressed as the relative standard deviation where applicable.

A matrix-matched certified reference material was not analyzed, and pre-digestion matrix-spike recovery experiments were not performed. Consequently, recovery values for the complete digestion and ICP-MS procedure are unavailable. Although the certified standard solution supported calibration traceability, it did not independently evaluate digestion efficiency or matrix-related effects in the coffee samples. This is recognized as a limitation of analytical validation.

### 2.5. Health Risk Assessment

#### 2.5.1. Estimated Daily Intake

The Estimated Daily Intake (EDI, mg/kg body weight per day) for each heavy metal was calculated as:

$$EDI=\frac{C\times IR}{BW}$$

where C is the mean heavy metal concentration in coffee (mg/kg d.w.), IR is the coffee intake rate (kg/day), and BW is average adult body weight (70 kg). The intake rate was derived from Saudi Arabia-specific consumption data: per capita coffee consumption of 5.39 kg/person/year [1], equating to IR = 14.77 g/day = 0.01477 kg/day. This represents a realistic chronic daily exposure estimate for Saudi adults.

#### 2.5.2. Non-Carcinogenic Risk Assessment

Non-carcinogenic risk was assessed using the Hazard Quotient (HQ) and Hazard Index (HI) approaches recommended by USEPA [22]:

$$HQ=\frac{EDI}{RfD}$$

where RfD is the USEPA oral reference dose (mg/kg/day) for each metal: Pb = 1.4 × 10^−3^; Cd = 1.0 × 10^−3^; As (inorganic) = 3.0 × 10^−4^; Cr(VI) = 3.0 × 10^−3^; Ni (soluble) = 2.0 × 10^−2^. An HQ < 1 indicates that non-carcinogenic risk is unlikely. The Hazard Index (HI) represents the sum of HQ values for all metals:

$$HI=\sum HQ_{i}$$

An HI > 1 indicates a potential cumulative non-carcinogenic health risk.

#### 2.5.3. Carcinogenic Risk Assessment

The Incremental Lifetime Cancer Risk (ILCR) was calculated for metals with established USEPA oral cancer slope factors (CSF) as:

$$ILCR=\frac{C\times IR\times EF\times ED\times CSF}{BW\times AT}$$

where EF is the exposure frequency (365 days/year), ED is the exposure duration (70 years), and AT is the lifetime averaging time for carcinogens (70 × 365 = 25,550 days). The oral cancer slope factors (CSFs; [mg/kg body weight/day]^−1^) used were 1.5 for As [23], 0.38 for Cd, and 0.0085 for Pb. Chromium was quantified as total Cr by ICP-MS, and no speciation analysis was performed to distinguish Cr(III) from Cr(VI). Therefore, a definitive chromium-specific ILCR could not be calculated. For screening purposes only, an upper-bound Cr-associated ILCR was estimated by conservatively assuming that 100% of the measured total Cr was present as Cr(VI) and applying the USEPA lifetime oral slope factor of 0.27 (mg/kg body weight/day)^−1^ for Cr(VI). This assumption represents a worst-case scenario and should not be interpreted as evidence that all chromium in the coffee samples was present as Cr(VI). Accordingly, the Cr-associated ILCR and the total ILCR including chromium were considered highly uncertain and were reported separately from the combined ILCR for As, Cd, and Pb. Nickel was excluded from the carcinogenic-risk calculation because its carcinogenic classification is primarily associated with inhalation exposure, and an appropriate oral cancer slope factor was not applied. For interpretation, an ILCR below 1 × 10^−6^ was considered negligible, values from 1 × 10^−6^ to 1 × 10^−4^ were considered within the commonly applied risk-management range, and values above 1 × 10^−4^ indicated a level warranting further evaluation.

#### 2.5.4. Statistical Analysis

Data were expressed as mean ± standard deviation (SD). The Shapiro–Wilk test was used to assess normality. Differences in metal concentrations across coffee types were evaluated using one-way ANOVA followed by Tukey’s honest significant difference (HSD) post hoc test. Pearson correlation coefficients were computed to examine inter-metal associations. Statistical analyses were performed using Python 3.11 (SciPy 1.12). Statistical significance was defined at *p* < 0.05.

Given the small number of samples within each category (*n* = 3), the statistical analyses were considered exploratory. An influential-observation sensitivity analysis was performed for the S-P3 nickel result of 70.687 mg/kg. Because no confirmed analytical or procedural failure was documented, this observation was not classified as an invalid measurement on the basis of its magnitude alone. Nickel descriptive statistics and population-level exposure estimates were therefore calculated under two scenarios: (i) excluding S-P3 to characterize the central distribution of the remaining samples, and (ii) including S-P3 to evaluate the influence of the observation on the study conclusions. Results from both scenarios are reported. Given the small sample size, no formal outlier-rejection test was used as the sole basis for removing the observation.

## 3. Results and Discussion

### 3.1. Heavy Metal Concentrations in Commercial Coffee Products

All five heavy metals were detected in all 21 coffee samples above the MDL (Method Detection Limit). Table 1 presents the mean ± SD concentrations (mg/kg d.w.) by coffee type, alongside the minimum and maximum values observed across the full sample set.

.

Lead concentrations varied most widely across sample types, with the dark roast category (D-P; 2.622 ± 2.639 mg/kg) and Yemeni coffee (YE; 2.548 ± 2.509 mg/kg) showing the highest mean values. Individual outliers within these categories were of particular concern: D-P2 recorded Pb = 5.662 mg/kg and YE-3 recorded 5.428 mg/kg concentrations approximately 56- and 54-fold above the Codex Alimentarius general food lead limit of 0.1 mg/kg, respectively. Blended coffee (B-P; 0.811 ± 0.402 mg/kg) exhibited the lowest mean lead concentrations.

Cadmium concentrations were consistently elevated across all sample types (range: 0.144–0.822 mg/kg d.w.), with all samples exceeding the EU maximum level for roasted coffee of 0.1 mg/kg (EU Regulation 2021/1323) by 1.4- to 8.2-fold. The blended product (B-P) showed the highest mean Cd (0.748 ± 0.066 mg/kg), followed by Ethiopian coffee (ET; 0.681 ± 0.062 mg/kg). Yemeni coffee showed the lowest Cd values (YE; 0.186 ± 0.072 mg/kg), though still exceeding the EU limit. Chromium concentrations ranged from 0.408 to 1.080 mg/kg, with Ethiopian coffee exhibiting the highest mean Cr (0.821 ± 0.234 mg/kg). Arsenic concentrations were comparatively low across all samples (range: 0.017–0.089 mg/kg), with Ethiopian coffee again showing the highest mean (0.048 ± 0.036 mg/kg).

Nickel concentrations ranged from 0.316 to 0.861 mg/kg in 20 samples, whereas S-P3 had a Ni concentration of 70.687 mg/kg, approximately 82-fold higher than the next-highest concentration. The elevated result was detected in both no-gas and He collision-cell acquisition modes, which indicates that it was not limited to one acquisition mode. However, repeat digestion, analysis of an independently prepared aliquot, and confirmation by an alternative analytical method were not performed. Therefore, the available evidence does not permit the result to be classified as either a confirmed analytical error or a confirmed product-specific contamination event.

A sensitivity analysis was consequently conducted. Excluding S-P3, the overall mean Ni concentration was 0.536 mg/kg, and the Saudi-packaged category mean was 0.443 mg/kg based on the two remaining samples. Including S-P3 increased the overall mean to approximately 3.876 mg/kg and the Saudi-packaged category mean to approximately 23.858 mg/kg. Thus, the Ni descriptive statistics, particularly those for the Saudi-packaged category, were highly sensitive to this single observation. Both scenarios are reported because the present data are insufficient to determine which provides a more representative estimate of the underlying product distribution.

### 3.2. Non-Carcinogenic Risk Assessment

Table 2 presents the estimated daily intake (EDI), hazard quotient (HQ), and cumulative hazard index (HI) for all five metals, calculated using Saudi Arabia-specific coffee consumption data (14.77 g/day).

The overall Hazard Index (HI = 0.438) was below the USEPA threshold of 1.0 for non-carcinogenic risk, indicating that, on a population-average basis, daily coffee consumption at the Saudi per capita rate does not exceed non-carcinogenic risk thresholds. However, this aggregate value masks substantial inter-sample variability. The dominant contributor to HI was lead (HQ_Pb = 0.247; 56.4% of total HI), followed by cadmium (HQ_Cd = 0.118; 26.9%), chromium (HQ_Cr = 0.044; 10.0%), arsenic (HQ_As = 0.024; 5.4%), and nickel (HQ_Ni = 0.006; 1.4%).

The sensitivity analysis showed that inclusion of S-P3 increased the mean Ni concentration from 0.536 to approximately 3.876 mg/kg and increased the Ni HQ from approximately 0.006 to 0.041. The corresponding total HI increased from 0.438 to approximately 0.473. Therefore, the S-P3 observation materially affected the Ni-specific exposure estimate but did not change the overall screening-level interpretation: the population-average HI remained below 1 under both scenarios. Nevertheless, the inclusion scenario should not be interpreted as evidence that all Saudi-packaged coffee products contain similarly elevated Ni concentrations, because it is based on one influential result within a category containing only three samples.

Notably, when HQ values for individual high-contamination samples are considered, the risk picture changes significantly. For D-P2 (Pb = 5.662 mg/kg), HQ_Pb alone reaches 0.854, meaning that a consumer exclusively drinking dark roast coffee of this batch would approach the non-carcinogenic threshold from lead alone. For YE-3 (Pb = 5.428 mg/kg), HQ_Pb = 0.819. When Cd, Cr, and As contributions are added for these samples, HI values of 1.10–1.15 are obtained, formally exceeding the non-carcinogenic risk threshold for consumers of these specific product batches.

### 3.3. Carcinogenic Risk Estimates and Interpretation

Table 3 presents the incremental lifetime cancer risks (ILCRs) associated with As, Cd, and Pb under lifetime exposure conditions based on the Saudi per capita coffee-consumption rate. Chromium was quantified as total Cr, and no speciation analysis was performed to distinguish Cr(III) from Cr(VI). Therefore, the chromium-associated ILCR shown in Table 3 is presented only as a conservative screening-level upper-bound estimate.

Chromium was quantified as total Cr, and no Cr(III)/Cr(VI) speciation analysis was performed. The Cr-associated ILCR was calculated solely as a conservative screening-level upper bound by assuming that 100% of the measured total Cr was present as Cr(VI) and applying the USEPA lifetime oral cancer slope factor of 0.27 (mg/kg-day)^−1^. This assumption does not indicate that all chromium in the analyzed coffee samples was present as Cr(VI). The resulting chromium-specific ILCR and the upper-bound total ILCR should therefore be interpreted with substantial caution. CSF: cancer slope factor; EF: exposure frequency, 365 days/year; ED: exposure duration, 70 years; AT: averaging time, 25,550 days; BW: body weight, 70 kg. An ILCR below 1 × 10^−6^ was considered negligible, whereas values from 1 × 10^−6^ to 1 × 10^−4^ were interpreted as being within the risk-management range applied in this study.

The USEPA’s 2024 IRIS assessment characterizes Cr(VI) as likely to be carcinogenic through oral exposure and provides a lifetime oral slope factor of 0.27 (mg/kg-day)^−1^. This value includes age-dependent adjustment factors and applies specifically to Cr(VI), not total chromium.

The combined ILCR for As, Cd, and Pb was 5.84 × 10^−5^. When the provisional upper-bound chromium contribution was included, the total ILCR increased to 9.38 × 10^−5^, which remained within, but approached the upper boundary of, the risk-management range applied in this study. Individual carcinogenic risks for As (1.06 × 10^−5^) and Pb (2.94 × 10^−6^) fell within the acceptable range (1 × 10^−6^ to 1 × 10^−4^).

### 3.4. Group-Level Lead Risk Comparison Across Coffee Types

The dark roast category (D-P) and Yemeni coffee (YE) carried the highest lead-associated risks, with HQ_Pb values of 0.395 and 0.384 respectively. Given that lead is also the dominant non-carcinogenic risk driver overall, these categories merit priority attention in any regulatory sampling programmed.

## 4. Discussion

### 4.1. Scale of Heavy Metal Contamination: Context and Regulatory Comparison

The most striking finding of the present study is the universal, systematic exceedance of the EU maximum level for cadmium in roasted coffee (0.1 mg/kg; EU Regulation 2021/1323) across all 21 samples, with mean Cd concentrations 5.6-fold above this threshold. Saudi Arabia does not currently enforce a specific MLs for cadmium in coffee, and our findings provide a strong evidence base for the Kingdom to align with international food safety standards through regulatory instruments under the Saudi Food and Drug Authority (SFDA). The EU ML reflects decades of EFSA scientific review acknowledging cadmium’s nephrotoxicity and established links to renal dysfunction, bone demineralisation (itai-itai disease), and carcinogenicity at the cadmium levels encountered in common dietary exposures. The fact that all coffee products analyzed in this study, including products of Brazilian origin and locally packaged qahwa blends, exceeded this comparison value indicates that the observed elevations were not restricted to a single product category. However, given the limited sample size and the absence of analyses of cultivation soils, irrigation water, processing equipment, packaging materials, and other potential contamination sources, the present findings cannot establish a systematic market-wide contamination problem or attribute the detected metals specifically to soil conditions [24].

Lead contamination was also alarming: mean concentrations of 1.638 mg/kg exceeded the general Codex Alimentarius food lead limit of 0.1 mg/kg by 16-fold on average, and by up to 57-fold in the worst individual samples (D-P2: 5.662 mg/kg; YE-3: 5.428 mg/kg). These extreme values within the dark roast and Yemeni categories likely reflect a combination of two effects: (i) geographic origin in mineral-rich volcanic soils (Yemen’s coffee-growing highlands overlie geologically active substrate), and (ii) roasting-induced concentration through mass loss.

The Pb concentrations exhibited very high relative standard deviations within the dark-roast and Yemeni coffee categories, with %RSD values of 100.6% and 98.5%, respectively. These values indicate substantial heterogeneity among the three samples analyzed within each category. Such variability is an important consideration when designing regulatory sampling programs because analysis of a single product batch could substantially overestimate or underestimate the typical concentration and corresponding consumer exposure. Nevertheless, given the small number of samples per category (n=3), these %RSD values should be interpreted as descriptive indicators of within-category variability rather than precise estimates of market-wide heterogeneity.

Chromium concentrations (range 0.408–1.080 mg/kg) and nickel concentrations (excluding the anomalous S-P3 outlier; range 0.316–0.861 mg/kg) reflect levels commonly reported in coffee products internationally and are consistent with natural soil background concentrations in tropical agricultural areas. The S-P3 Ni concentration of 70.687 mg/kg was markedly higher than all other Ni measurements and exerted a substantial influence on the Saudi-packaged category mean and the overall Ni exposure estimate. Although contamination from grinding, processing, or packaging equipment is one plausible explanation, the present study did not investigate these sources, and the available analytical evidence does not establish the origin of the elevated value. It could represent a genuine sample-specific contamination event, contamination introduced during preparation or handling, or another unrecognized analytical event. Confirmatory analysis would require repeat digestion of a retained sample, analysis of an independently prepared aliquot, examination of an independently purchased package or batch, and, where feasible, confirmation using an alternative validated analytical method. Arsenic concentrations (mean 0.034 mg/kg), while relatively low in absolute terms, still merit consideration given arsenic’s potent diabetogenic and carcinogenic properties at even low chronic exposures.

### 4.2. Alternative and Rapid Methods for Heavy-Metal Detection

In addition to ICP-MS, several analytical techniques can be used to detect and quantify heavy metals in food products. Atomic absorption spectrometry, particularly graphite-furnace atomic absorption spectrometry, provides sensitive targeted analysis of individual metals and may be more accessible to laboratories without ICP-MS facilities. Inductively coupled plasma optical emission spectrometry enables simultaneous multi-element analysis, although its detection limits are generally higher than those of ICP-MS. X-ray fluorescence spectroscopy offers rapid and minimally destructive elemental screening of solid or powdered food samples with limited sample preparation. However, its sensitivity and quantitative performance may be affected by matrix composition, particle size, moisture content, sample homogeneity, and the availability of suitable matrix-matched calibration materials. Electrochemical sensors, colorimetric assays, fluorescence-based sensors, and paper-based devices are also being developed for portable and relatively low-cost food-safety screening [25].

Surface-enhanced Raman spectroscopy represents a particularly promising approach for the rapid screening of heavy metals in food and agricultural products. SERS uses nanostructured plasmonic substrates, commonly based on gold, silver, or composite nanomaterials, to enhance Raman signals. Because free metal ions generally do not produce sufficiently strong and distinctive Raman spectra, their detection commonly relies on selective recognition systems, including functionalized nanoparticles, chelating ligands, aptamers, ion-imprinted polymers, aggregation probes, or Raman reporter molecules. SERS-based approaches have been investigated for detecting Pb, Cd, As, Hg, Cr, and other metals and metalloids in food and agricultural matrices. The main potential advantages of SERS include rapid data acquisition, high sensitivity, small sample-volume requirements, and compatibility with portable Raman instruments [26,27].

Nevertheless, SERS does not completely eliminate the need for sample preparation or analytical validation. Its performance in complex food matrices may be affected by substrate-to-substrate variability, uneven distribution of signal-enhancing hot spots, nanoparticle aggregation, competing ions, nonspecific adsorption, extraction efficiency, and interference from matrix constituents. Reliable quantitative use may therefore require reproducible extraction and matrix-cleanup procedures, standardized substrates, matrix-matched calibration, chemometric analysis, and validation against a recognized reference method. These limitations are particularly relevant to commercial coffee products containing compositionally different matrices, such as coffee solids, cardamom, sugar, or other added ingredients [26,27].

For coffee surveillance, SERS could be used as a complementary rapid-screening method to identify products requiring further investigation, particularly in settings with limited access to centralized analytical facilities. However, validated ICP-MS or another established elemental-analysis technique would remain necessary for confirmatory multi-element quantification, regulatory comparison, and calculation of dietary exposure and health-risk indices. A tiered monitoring strategy could therefore combine portable SERS or other sensor-based methods for preliminary screening with confirmatory ICP-MS analysis of samples producing elevated, positive, or uncertain results. Future studies should compare SERS and ICP-MS directly in coffee beans, composite coffee products, and brewed beverages and evaluate detection limits, selectivity, recovery, reproducibility, matrix effects, and variation among SERS substrate batches [28].

### 4.3. Mechanistic Links Between Coffee-Associated Heavy Metal Exposure and Type 2 Diabetes

The relationship between heavy metal contamination in coffee and diabetes risk must be understood through multiple converging mechanistic pathways. As outlined in the Introduction, the individual metals detected in the present study are each supported by varying degrees of epidemiological and mechanistic evidence as diabetogenic agents. Here, we integrate these pathways with the specific contamination profile observed [29].

#### 4.3.1. Cadmium and Pancreatic β-Cell Dysfunction

Cadmium is the best characterized diabetogenic heavy metal and the one present at the most consistently alarming concentrations in our dataset. Mechanistically, Cd^2+^ ions are taken up into pancreatic β-cells via calcium channels and ZIP8/ZIP14 transporters (shared with the essential micronutrient zinc), where they exert multiple toxic effects: (i) competitive displacement of Zn^2+^ from the crystalline insulin hexamer structure, disrupting insulin folding, packaging into secretory granules, and zinc-dependent processing by proinsulin convertases; (ii) induction of mitochondrial dysfunction through inhibition of the electron transport chain complexes I, II, and III, reducing ATP availability for calcium-dependent insulin exocytosis; (iii) generation of reactive oxygen species leading to oxidative DNA damage and activation of apoptotic pathways (cytochrome c release, caspase-3 activation) that reduce β-cell mass over time; (iv) upregulation of pro-inflammatory cytokines (IL-6, TNF-α, IL-1β) in surrounding pancreatic tissue, establishing a chronic low-grade inflammatory microenvironment that further impairs β-cell function and promotes peripheral insulin resistance; and (v) inhibition of the PI3K/Akt/mTOR signalling pathway downstream of the insulin receptor in hepatocytes and skeletal muscle, directly impairing glucose uptake and glycogen synthesis [29,30].

The prospective Wuhan-Zhuhai cohort study (*n* = 3521) demonstrated that each one-unit increase in log-transformed urinary cadmium was associated with a 42% increase in prevalent type 2 diabetes risk (OR = 1.42; 95% CI: 1.16–1.73), and a Pakistani case–control study (*n* = 724) found that Cd-exposed participants had significantly elevated HbA1c, random blood glucose, serum α-amylase, DPP-IV, and IL-6 levels, confirming both chronic glycaemic deterioration and pancreatic injury in vivo. The chronic daily EDI for Cd from coffee alone in the present study (1.18 × 10^−4^ mg/kg/day) represents 11.8% of the USEPA oral RfD, a non-trivial fraction and must be considered in the context of total dietary Cd exposure from rice, vegetables, and other sources that commonly reach or exceed the JECFA Provisional Tolerable Monthly Intake (PTMI) of 25 μg/kg BW/month in populations with high rice consumption [31].

#### 4.3.2. Lead, Insulin Signaling, and Cardiovascular Risk

Lead emerged as the primary driver of non-carcinogenic risk in our assessment (HQ_Pb = 0.247; 56% of total HI), and mechanistically it exerts diabetogenic effects through a distinct pathway from cadmium. Lead preferentially accumulates in bone (with a biological half-life of decades), from which it is remobilized during periods of bone resorption (osteoporosis, pregnancy, ageing), creating chronic low-level circulating Pb^2+^ exposure even after dietary exposure ceases. In pancreatic tissue, lead generates ROS that disrupt calcium homeostasis, calcium being the essential second messenger that triggers insulin vesicle fusion with the plasma membrane. Lead additionally modulates protein kinase C (PKC) activity by mimicking the calcium-mediated activation of this enzyme, leading to aberrant phosphorylation of the insulin receptor substrate-1 (IRS-1) at serine residues rather than tyrosine, effectively switching IRS-1 from an insulin-signaling activator to an inhibitor a molecular mechanism of insulin resistance [32,33].

Beyond its diabetogenic effects, lead poses substantial cardiovascular risk relevant to the Saudi context. Lead exposure is a well-established cause of hypertension through multiple mechanisms: inhibition of Na^+^/K^+^-ATPase in vascular smooth muscle (increasing vascular tone), impairment of endothelial nitric oxide synthase (eNOS) and consequent reduction in vasorelaxatory NO production, and stimulation of the renin–angiotensin–aldosterone system. Epidemiological data consistently link blood lead levels with increased risk of myocardial infarction, stroke, and all-cause cardiovascular mortality. In Saudi Arabia, where hypertension prevalence is estimated at 25–35% of the adult population, additional lead exposure from contaminated dietary sources, including coffee, may contribute meaningfully to population-level cardiovascular risk [33].

#### 4.3.3. Arsenic and Glucose Homeostasis

Although arsenic concentrations in our dataset were relatively low (mean 0.034 mg/kg; HQ_As = 0.024), arsenic’s diabetogenic potency demands consideration even at low exposure levels. Inorganic arsenic (iAs), the relevant carcinogenic and diabetogenic species, is taken up into cells via aquaporin channels and phosphate transporters. Intracellularly, iAs^3+^ inhibits pyruvate dehydrogenase (PDH) and α-ketoglutarate dehydrogenase by binding to lipoic acid cofactors, disrupting the TCA cycle and shifting glucose metabolism toward inefficient anaerobic glycolysis in β-cells. This reduces ATP production and impairs calcium-dependent insulin secretion. Arsenic also induces endoplasmic reticulum (ER) stress in β-cells through activation of the unfolded protein response (UPR), leading to apoptotic cell death via CHOP/GADD153 signaling. The SWAN prospective cohort’s finding of a 19% increase in diabetes risk per doubling of urinary arsenic (HR = 1.19; 95% CI: 1.10–1.30) over 16 years, at exposure levels comparable to or lower than those implied by our coffee data, underscores the relevance of this exposure pathway [19].

#### 4.3.4. Nickel and Chromium

The concentrations of Ni and Cr reported in the present study were measured in dry coffee products before brewing or contact with preparation and serving vessels. These concentrations therefore represent metals already present in the commercial coffee material and may reflect contributions from one or more stages, including cultivation, harvesting, drying, roasting, grinding, processing, packaging, storage, sample preparation, or laboratory handling. However, the present study was not designed to identify or differentiate among these potential sources.

The metal content of the consumed beverage may differ from that measured in the dry coffee. During preparation, only a fraction of the metals present in the dry material may be extracted into the beverage, while additional contributions could potentially arise from brewing water, additives, grinding equipment, or contact with preparation and serving vessels. Nickel and chromium are constituents of stainless-steel alloys, and metal migration from cookware may be influenced by material composition, surface condition, repeated use, heating duration, and contact conditions. Traditional Arabic coffee preparation may involve dallah vessels of different materials, ages, and surface conditions. Therefore, these vessels could represent an additional source of metal migration that was not captured by the dry-coffee analysis. Nevertheless, because neither brewed coffee nor dallah vessels were analyzed in the present study, their contribution to the final metal concentrations and consumer exposure cannot be determined from the current results.

Aluminum migration from brewing vessels may also be relevant to neurological health. Experimental evidence indicates that aluminum can promote oxidative stress, neuroinflammation, mitochondrial dysfunction, amyloid-β aggregation, and tau phosphorylation, which are processes implicated in Alzheimer’s disease. However, epidemiological evidence remains inconsistent, and a causal relationship has not been established. Aluminum should therefore be regarded as a possible contributing factor rather than a confirmed cause of Alzheimer’s disease [Reference]. Because aluminum was not quantified in the present study, no conclusions can be drawn regarding aluminum exposure from the analyzed products or prepared coffee beverages [34,35,36].

Sample S-P3 had a Ni concentration of 70.687 mg/kg, substantially exceeding the values measured in the other samples. Because there was no documented analytical or procedural failure, the result was retained as an unverified, influential observation and evaluated through sensitivity analysis rather than being treated as a confirmed error. Excluding S-P3 produced an overall mean Ni concentration of 0.536 mg/kg, a Ni HQ of approximately 0.006, and a total HI of 0.438. Including S-P3 increased the corresponding values to approximately 3.876 mg/kg, 0.041, and 0.473, respectively (Table 1 and Table 2). Inclusion therefore changed the magnitude of the Ni-specific estimate but did not change the overall screening-level conclusion that the population-average HI was below 1.

At the category level, however, inclusion of S-P3 increased the Saudi-packaged mean from 0.443 mg/kg, based on two samples, to approximately 23.858 mg/kg, based on three samples. The extreme sensitivity of this estimate and the limited category sample size prevent reliable generalization to Saudi-packaged coffee products. The observation should instead be considered a signal requiring targeted confirmatory sampling and analysis. Elevated Ni exposure has been associated with altered glucose metabolism in experimental animal studies through increased hepatic gluconeogenesis, reduced peripheral glucose utilization, and changes in pancreatic function [37]. The EFSA TDI for nickel of 13 μg/kg BW/day provides context: if the S-P3 Ni level were representative of the entire S-P category, a consumer ingesting 14.77 g/day would have a Ni EDI of 14.95 μg/kg/day, exceeding the EFSA TDI [38]. This hypothetical scenario underscores the urgent need for monitoring Saudi-packaged coffee products for nickel contamination from processing equipment.

Hexavalent chromium Cr(VI), the carcinogenic species, is classified as a Group 1 human carcinogen (IARC) primarily through mutagenic and oxidative DNA damage in the nucleus. Trivalent Cr(III), in contrast, is an essential micronutrient that potentiates insulin signaling by facilitating the binding of insulin to its receptor [39]. The differential health impact of chromium in coffee therefore depends critically on the Cr speciation information, which is not available from total Cr ICP-MS measurements and would require species-specific analysis (e.g., ion chromatography-ICP-MS). This represents a key limitation of the present study and an important direction for future investigation.

#### 4.3.5. Roasting Degree as a Modulator of Heavy Metal Risk and Protective Compound Loss

The data in Table 1 and Table 4 demonstrate that dark roast coffee (D-P) carries the highest mean lead concentrations (2.622 mg/kg) of all seven sample categories, consistent with the roasting-induced concentration effect described in the Introduction. During dark roasting, approximately 20–30% of the bean’s total mass is lost as water vapour and volatile organic compounds, while non-volatile metals are retained in the solid matrix. This mass-loss concentration effect means that the same green bean, when dark-roasted compared to light-roasted, will yield a higher metal concentration per unit mass of the final product.

This toxic metal concentration effect occurs simultaneously with progressive destruction of chlorogenic acids (CGAs), the primary bioactive antioxidants in coffee responsible for much of coffee’s observed metabolic protection. CGAs comprise 6–12% of green coffee dry weight but may be reduced to 1–2% in dark espresso roasts. CGAs modulate glucose metabolism through multiple mechanisms: inhibition of glucose-6-phosphatase (reducing hepatic glucose output), promotion of GLP-1 secretion from intestinal L-cells (thereby stimulating insulin secretion), and direct antioxidant protection of β-cells. The progressive loss of these protective compounds with increasing roast depth means that darker roasts offer less metabolic protection while simultaneously delivering higher heavy metal loads—a doubly unfavorable trade-off for consumers at metabolic risk.

The higher lead levels in Yemeni coffee (YE; 2.548 mg/kg mean) likely reflect a distinct mechanism: the volcanic geology of Yemen’s highland coffee-producing regions (particularly the Haraz, Bani Matar, and Haymah districts) is associated with naturally elevated soil lead and mineral content. Traditional Yemeni coffee processing practices including natural sun-drying on bare soil surfaces, use of unlined clay or metal fermentation vessels, and extended dry milling may also introduce exogenous metal contamination during post-harvest processing, independently of soil content.

### 4.4. Saudi Coffee (Arabic Coffee or Qahwa) Preparation: A Unique Risk Context

The Arabic/cardamom coffee category (CA-P) warrants dedicated discussion given its unique cultural importance in Saudi Arabia and its distinct preparation method. Qahwa is typically prepared from lightly roasted green or pale-yellow coffee beans (*Coffea arabica*) combined with ground cardamom (*Elettaria cardamomum*), and in some regional preparations, saffron and cloves. Unlike espresso or filter coffee, qahwa is brewed in a dallah pot directly over a flame or on a stove burner, with the grounds remaining in contact with the liquid for extended periods (typically 15–30 min) and the beverage is often kept warm and reheated multiple times over several hours during social gatherings.

The dallah pot itself is therefore a critical, undercharacterised metal contamination source. Traditional dallah pots are made from copper, brass (copper-zinc alloy), or aluminum; modern commercial versions may be stainless steel or silver-plated. Copper and brass surfaces, particularly when unlined or when the protective patina has been disrupted by cleaning with acidic substances (a common practice), leach Cu^2+^ and Zn^2+^ ions into hot acidic coffee brew. While copper and zinc at trace levels are essential micronutrients, at elevated concentrations copper can inhibit antioxidant enzymes and zinc in excess can paradoxically impair β-cell function. Aluminum dallah pots pose a distinct concern: Al^3+^ ions are released into hot acidic brews at rates of 1–4 mg/L in published studies, and aluminum toxicity has been proposed as a promoter of insulin resistance through interference with insulin receptor substrate phosphorylation and promotion of amyloid β aggregation in pancreatic tissue.

The present study found that CA-P coffee had a mean Pb concentration of 1.525 mg/kg and a mean Cd concentration of 0.572 mg/kg, both within the range of the other commercial coffee categories. However, these measurements were made on dry bean/ground coffee samples, not on brewed qahwa beverage. The brewed metal concentrations could differ substantially depending on brewing temperature, contact time, water hardness, dallah composition, and the presence of cardamom (which itself may contribute trace metals depending on its origin and processing). A dedicated study of metal leaching during traditional dallah-based qahwa preparation is an important future research priority for public health in Saudi Arabia and the broader GCC.

### 4.5. Coffee Consumption, Diabetes, and Cardiovascular Disease: Reconciling the Evidence

A central paradox in the literature on coffee and diabetes is that large epidemiological cohort studies consistently report an inverse, dose-dependent association between habitual coffee consumption and long-term type 2 diabetes risk, with each additional cup per day associated with approximately a 6–8% reduction in relative risk in meta-analyses [40]. This apparently protective relationship has been attributed to the bioactive non-caffeine constituents of coffee, particularly chlorogenic acids, trigonelline, magnesium, and anti-inflammatory polyphenols, which collectively improve insulin sensitivity, reduce hepatic glucose output, modulate GLP-1 secretion, and protect β-cells from oxidative stress [41].

The present data do not contradict these epidemiological findings but add an important caveat: the cohort studies generating evidence of coffee’s protective effects (Nurses’ Health Study, Health Professionals Follow-up Study, European Prospective Investigation into Cancer and Nutrition [EPIC]) were primarily conducted in populations consuming filtered drip coffee from commercially regulated food markets in North America and Western Europe markets with substantially more rigorous enforcement of heavy metal maximum limits than is currently the case in Saudi Arabia. In these populations, the health benefits of coffee’s bioactive compounds may genuinely outweigh the risks from the relatively low heavy metal concentrations permitted by strict regulatory standards [42].

In Saudi Arabia, the equation may be fundamentally different: consumers are exposed to heavy metal concentrations in coffee that universally exceed EU Cd limits and substantially exceed general Codex Pb limits. When cadmium concentrations are 5–8 times the EU ML, and when the brewed dallah preparation method may add further metal leaching from the vessel, the protective bioactive compounds may be insufficient to offset the pancreatic, hepatic, and vascular toxicity being simultaneously imposed by the heavy metal load. The dose–response relationship for coffee and diabetes risk may therefore be non-monotonic in populations with high-contamination exposure: moderate consumption of low-contamination coffee may be protective, while equivalent consumption of highly contaminated coffee may be neutral or harmful [30,43].

This hypothesis is supported by the mechanistic literature: chlorogenic acids at typical concentrations in freshly brewed coffee provide antioxidant protection equivalent to approximately 500–2000 nmol Trolox equivalents per cup. The oxidative burden imposed by 0.56 mg/kg cadmium generating sustained hydroxyl radical production through Fenton-like reactions may exceed the antioxidant buffering capacity provided by CGAs, particularly in dark roasts where CGA content is already substantially reduced. This mechanistic calculation underscores the need for studies designed specifically to assess coffee’s net metabolic impact in populations where heavy metal contamination is high, rather than extrapolating from regulatory-compliant market populations [43].

### 4.6. Carcinogenic Risk: Public Health Implications for the Saudi Population

The combined ILCR for As, Cd, and Pb was $5.84\times 10^{-5}$, which was within the risk-management range of 1 × 10^−6^ to 1 × 10^−4^ applied in this study. When a provisional chromium contribution was included, the upper-bound total ILCR increased to $9.38\times 10^{-5}$. Although this value remained within the applied range, it approached its upper boundary and therefore warrants careful interpretation.

The upper-bound total ILCR of 9.38 × 10^−5^ did not exceed the upper boundary of 1 × 10^−4^ applied in this study, although it approached this value and therefore warrants careful interpretation. An ILCR of 1 × 10^−4^ conventionally represents an estimated upper-bound probability of one additional cancer case per 10,000 similarly exposed individuals over a lifetime. However, the present screening-level estimate should not be extrapolated directly to predict the number of cancer cases in the Saudi population because the study included only 21 dry-coffee samples and did not measure metal extraction during brewing, gastrointestinal bioaccessibility, individual consumption patterns, or additional preparation-related sources of exposure [44].

Cadmium was the largest quantified contributor to the upper-bound total ILCR, accounting for approximately 47.9%, followed by the provisional chromium contribution at 37.7%, arsenic at 11.3%, and lead at 3.1%. Cadmium and Cr(VI) are recognized carcinogenic hazards, but the chromium-related result in the present study is subject to substantial uncertainty. Chromium was measured as total Cr by ICP-MS, and no speciation analysis was performed to distinguish Cr(III) from Cr(VI). The oral cancer slope factor of 0.27 ${\left(mg/kg\;body\;weight/day\right)}^{-1}$ used in the screening calculation applies specifically to Cr(VI), not to total chromium. The Cr-associated ILCR of $3.54\times 10^{-5}$ was therefore calculated solely as a conservative upper-bound estimate by assuming that 100% of the measured total Cr was present as Cr(VI). This assumption is not evidence that Cr(VI) was present at the measured total chromium concentration and may substantially overestimate the actual chromium-associated risk. Consequently, the chromium estimate should not be interpreted as a definitive carcinogenic-risk value or as evidence that chromium was a confirmed carcinogenic-risk driver. Species-specific chromium analysis, preferably using a validated separation technique such as ion chromatography coupled with ICP-MS, is required to quantify Cr(VI) and support a reliable chromium-specific cancer-risk assessment. The USEPA oral cancer slope factor applies specifically to Cr(VI), whereas the present ICP-MS measurements represent total chromium.

### 4.7. Limitations of the Study

Several limitations should be acknowledged. First, the study included only 21 products, with three independently purchased packages or batches from each of seven coffee categories. This limited sample size reduces the statistical power to detect inter-category differences and does not capture the full diversity of brands, geographic origins, roasting levels, production batches, packaging types, and product formulations available in the Saudi market. The findings should therefore be regarded as exploratory and limited to the products and batches analyzed.

Second, the analysis was performed on dry coffee products rather than brewed beverages. The fractions of metals extracted during brewing and subsequently available for gastrointestinal absorption may vary according to the metal, brewing method, temperature, contact time, pH, water composition, and coffee-to-water ratio. Therefore, the concentrations measured in the dry products should not be interpreted as equivalent to concentrations in consumed beverages or as direct estimates of absorbed doses. Possible contributions from brewing water, additives, grinding equipment, and preparation or serving vessels, particularly those used for traditional qahwa, were also not evaluated.

Third, chromium was quantified as total Cr, and no speciation analysis was performed to determine the relative proportions of Cr(III) and Cr(VI). This distinction is important because the oral cancer slope factor used in the screening assessment applies specifically to Cr(VI). Consequently, the Cr-associated ILCR, calculated by assuming that all measured Cr was present as Cr(VI), represents a conservative upper-bound estimate and may substantially overestimate the actual risk. A definitive chromium-specific risk assessment would require species-specific analysis using a validated separation method, such as ion chromatography coupled with ICP-MS.

Fourth, a matrix-matched certified reference material was not analyzed, and pre-digestion matrix-spike recovery experiments were not conducted. Recovery values for the complete digestion and ICP-MS procedure were therefore unavailable, and possible effects associated with digestion efficiency, analyte loss, and the coffee matrix could not be independently evaluated. Although a certified multi-element standard solution supported instrument-calibration traceability, it did not provide complete validation of the digestion and analytical procedure.

An additional limitation concerns the influential S-P3 Ni result. Although the elevated concentration was detected in both no-gas and He collision-cell acquisition modes, repeated digestion of the retained sample, analysis of an independently prepared aliquot, confirmation using an alternative method, and analysis of another package from the same batch were not performed. It was therefore not possible to determine whether the result represented genuine sample-specific contamination or a preparation-related or analytical event. The sensitivity analysis quantified its influence on the descriptive and risk estimates but could not establish its origin or reproducibility. Because the Saudi-packaged category contained only three samples, neither the estimate including S-P3 nor that excluding S-P3 should be considered a stable estimate of the wider product category.

Finally, the risk assessment did not account for background exposure to the investigated metals from other dietary and environmental sources, such as food and drinking water, and therefore does not represent total cumulative exposure. Individual differences in coffee consumption, body weight, gastrointestinal bioaccessibility, lifestyle, health status, and susceptibility to metal toxicity were also not incorporated. Potential variation associated with genetic polymorphisms affecting metal transport, metabolism, and detoxification, including metallothionein, glutathione S-transferase, and ABCC2 transporters, was outside the scope of this screening-level assessment [45].

## 5. Conclusions

This exploratory study detected Pb, Cd, As, Cr, and Ni in 21 commercial coffee products marketed in Saudi Arabia. The overall non-carcinogenic hazard index based on mean concentrations remained below 1, with Pb and Cd as the principal contributors. The combined ILCR for As, Cd, and Pb was within the risk-management range applied in this study. The chromium-associated estimate was highly uncertain because total Cr, rather than Cr(VI), was measured.

The S-P3 observation substantially influenced the Ni-specific descriptive and exposure estimates. Including S-P3 increased the overall mean Ni concentration from 0.536 to 3.876 mg/kg and the total HI from 0.438 to 0.473, but it did not alter the overall screening-level interpretation. Nevertheless, the origin and reproducibility of this observation remain unresolved and require confirmatory analysis.

The findings of this exploratory study are consistent with previous reports of heavy-metal contamination in coffee from Saudi Arabia and other countries and therefore contribute to a broader international evidence base. The potential relevance of the detected metals to diabetes and cardiovascular disease is supported by published toxicological, mechanistic, and epidemiological evidence. However, because clinical biomarkers and disease outcomes were not evaluated, the present study identifies contaminated coffee as a potential dietary exposure pathway rather than independently establishing a causal relationship with these diseases. Larger and analytically validated studies of both dry and brewed coffee are needed to strengthen market-level exposure estimates.

## Figures and Tables

**Table 1:** (**A**). Mean ± SD concentrations of heavy metals (mg/kg d.w.) in seven categories of commercial coffee products from the Saudi Arabian market, measured by ICP-MS. (**B**). Sensitivity analysis of nickel concentrations in commercial coffee samples, showing the Saudi-packaged category mean, overall mean, and overall range with and without the influential S-P3 observation. Concentrations are expressed as mg/kg dry weight.

**A**					
**Coffee Type (*n* = 3)**	**Cr (mg/kg)**	**Ni (mg/kg)**	**As (mg/kg)**	**Cd (mg/kg)**	**Pb (mg/kg)**
MDL (mg/kg d.w.)	0.00353	0.00722	0.00153	0.000224	0.000375
Brazilian (Br)	0.703 ± 0.150	0.523 ± 0.036	0.035 ± 0.012	0.569 ± 0.049	1.152 ± 0.790
Ethiopian (ET)	0.821 ± 0.234	0.787 ± 0.119	0.048 ± 0.036	0.681 ± 0.062	1.405 ± 0.465
Saudi-packaged (S-P)	0.541 ± 0.004	0.443 ± 0.023 * or 23.858	0.023 ± 0.004	0.571 ± 0.216	1.403 ± 0.812
Blend Product (B-P)	0.521 ± 0.074	0.389 ± 0.047	0.028 ± 0.016	0.748 ± 0.066	0.811 ± 0.402
Dark Roast (D-P)	0.596 ± 0.064	0.563 ± 0.034	0.033 ± 0.020	0.595 ± 0.066	2.622 ± 2.639
Yemeni (YE)	0.455 ± 0.055	0.377 ± 0.067	0.044 ± 0.008	0.186 ± 0.072	2.548 ± 2.509
Arabic/Cardamom (CA-P)	0.719 ± 0.231	0.638 ± 0.214	0.024 ± 0.005	0.572 ± 0.115	1.525 ± 0.796
Overall Mean ± SD	0.622 ± 0.172	0.536 ± 0.165 *	0.034 ± 0.017	0.560 ± 0.192	1.638 ± 1.408
Min–Max	0.408–1.080	0.316–0.861 *	0.017–0.089	0.144–0.822	0.519–5.662
EU ML/Regulatory Limit	No specific ML	No specific ML	No specific ML	0.1 mg/kg †	0.1 mg/kg ‡
**B**					
**Nickel Result**		**Excluding S-P3**		**Include S-P3**	
Saudi-packaged category mean, mg/kg		0.443		23.858	
Overall mean, mg/kg		0.536		3.876	
Overall range, mg/kg		0.316 to 0.861		0.316 to 70.687	

* Sample S-P3 had a Ni concentration of 70.687 mg/kg, approximately 82-fold higher than the next-highest Ni concentration. The result was detected in both no-gas and He collision-cell acquisition modes; however, repeat digestion, analysis of an independently prepared aliquot, and confirmation using a second analytical method were not performed. Consequently, the result could not be confirmed as either a true sample-specific concentration or an analytical or preparation-related event. Nickel results are therefore presented using two sensitivity-analysis scenarios: excluding S-P3 and including S-P3. The exclusion scenario describes the distribution of the remaining samples but should not be interpreted as proof that S-P3 was analytically invalid. † EU Regulation 2021/1323 (roasted coffee). ‡ Codex Alimentarius general food ML (CAC/GL 5-1989, Rev. 4). MDL: method detection limit; d.w.: dry weight. The MDLs were converted from the solution detection limits to a sample-mass basis using a final digest volume of 0.050 L and a nominal sample mass of 0.001 kg. Accordingly, $MDL\;\;(mg/kg)={DL}_{solution}\;\;(\mu g/L)\times 0.050$.

**Table 2:** (**A**) Estimated Daily Intake (EDI), Hazard Quotient (HQ), and Hazard Index (HI) for heavy metals from coffee consumption by Saudi adults. (**B**) Sensitivity analysis of the influence of sample S-P3 on nickel exposure and non-carcinogenic risk estimates, showing the mean Ni concentration, estimated daily intake (EDI), Ni hazard quotient (HQ), and total hazard index (HI) under exclusion and inclusion scenarios.

**A**						
**Parameter**	**Cr**	**Ni**	**As**	**Cd**	**Pb**	**HI (Sum)**
Mean conc. (mg/kg)	0.622	0.536	0.034	0.560	1.638	—
EDI (mg/kg/day)	1.31 × 10^−4^	1.13 × 10^−4^	7.07 × 10^−6^	1.18 × 10^−4^	3.46 × 10^−4^	—
RfD (mg/kg/day)	3.0 × 10^−3^	2.0 × 10^−2^	3.0 × 10^−4^	1.0 × 10^−3^	1.4 × 10^−3^	—
HQ	0.044	0.006	0.024	0.118	0.247	0.438
Risk Level	Safe	Safe	Safe	Safe	Safe	Safe (<1)
**B**						
**Scenario**	**Mean Ni Concentration (mg/kg)**		**Ni EDI (mg/kg BW/day)**		**Ni HQ**	**Total HI**
Excluding S-P3	0.536		1.13 × 10^−4^		0.006	0.438
Including S-P3	3.876		8.18 × 10^−4^		0.041	0.473

EDI: Estimated Daily Intake; RfD: oral Reference Dose (USEPA IRIS); HQ: Hazard Quotient; HI: Hazard Index. IR = 14.77 g coffee/day; BW = 70 kg. Calculations used IR = 0.01477 kg coffee/day, BW = 70 kg, and an oral Ni reference dose of 2.0 × 10^−2^ mg/kg BW/day. The total HI for the inclusion scenario was calculated by replacing the Ni contribution in the original analysis with the Ni HQ derived from the mean concentration including S-P3. Minor differences may arise from rounding.

**Table 3:** Incremental lifetime cancer risk associated with exposure to metals through coffee consumption in Saudi Arabia.

Metal	Mean Conc. (mg/kg)	CSF (mg/kg/day)^−1^	ILCR	Classification
As	0.034	1.5	1.06 × 10^−5^	Within acceptable range
Cd	0.560	0.38	4.49 × 10^−5^	Within acceptable range
Cr, upper-bound screening estimate only *	0.622 total Cr	0.27 for Cr(VI)	3.54 × 10^−5^	Highly uncertain upper-bound estimate
Pb	1.638	0.0085	2.94 × 10^−6^	Within acceptable range
Combined ILCR excluding Cr	—	—	5.84 × 10^−5^	Within the applied risk range
Upper-bound total ILCR including provisional Cr estimate	—	—	9.38 × 10^−5^	Within, but close to the upper boundary of, the applied risk range

* Conservative assumption: 100% of detected Cr is Cr(VI); actual Cr(VI) fraction is typically lower, which would reduce ILCR_Cr proportionally. CSF: Cancer Slope Factor (USEPA IRIS). EF = 365 days/year; ED = 70 years; AT = 25,550 days; BW = 70 kg. USEPA acceptable ILCR range: 1 × 10^−6^ to 1 × 10^−4^.

**Table 4:** Group-level Pb-specific EDI, HQ, and ILCR by coffee category.

Coffee Category	Mean Pb (mg/kg)	EDI_Pb (mg/kg/day)	HQ_Pb	ILCR_Pb	Relative Risk
Blend Product (B-P)	0.811	1.71 × 10^−4^	0.122	1.46 × 10^−6^	Lowest
Brazilian (Br)	1.152	2.43 × 10^−4^	0.174	2.07 × 10^−6^	Low
Saudi-packaged (S-P)	1.403	2.96 × 10^−4^	0.211	2.52 × 10^−6^	Moderate
Ethiopian (ET)	1.405	2.96 × 10^−4^	0.212	2.52 × 10^−6^	Moderate
Arabic/Cardamom (CA-P)	1.525	3.22 × 10^−4^	0.230	2.74 × 10^−6^	Moderate
Yemeni (YE)	2.548	5.38 × 10^−4^	0.384	4.57 × 10^−6^	High
Dark Roast (D-P)	2.622	5.53 × 10^−4^	0.395	4.70 × 10^−6^	Highest

## Data Availability

The raw data supporting the conclusions of this article will be made available by the authors on request.

## Abbreviations

The following abbreviations are used in this manuscript:

As	Arsenic
AT	Averaging Time
BW	Body Weight
CA-P	Arabic/Cardamom Coffee Product (Qahwa)
Cd	Cadmium
CGAs	Chlorogenic Acids
Cr	Chromium
Cr(VI)	Hexavalent Chromium
Cr(III)	Trivalent Chromium
CSF	Cancer Slope Factor
DALYs	Disability-Adjusted Life Years
EDI	Estimated Daily Intake
EF	Exposure Frequency
ED	Exposure Duration
eNOS	Endothelial Nitric Oxide Synthase
EPA	Environmental Protection Agency
EU	European Union
GCC	Gulf Cooperation Council
HI	Hazard Index
HQ	Hazard Quotient
ICP-MS	Inductively Coupled Plasma Mass Spectrometry
IDF	International Diabetes Federation
ILCR	Incremental Lifetime Cancer Risk
IR	Intake Rate
JECFA	Joint FAO/WHO Expert Committee on Food Additives
MENA	Middle East and North Africa
MDL	Method Detection Limit
NF-κB	Nuclear Factor Kappa B
Ni	Nickel
NCD	Non-Communicable Disease
Pb	Lead
PI3K	Phosphoinositide 3-Kinase
QA/QC	Quality Assurance/Quality Control
RfD	Reference Dose
ROS	Reactive Oxygen Species
SD	Standard Deviation
SFDA	Saudi Food and Drug Authority
T2DM	Type 2 Diabetes Mellitus
TNF-α	Tumor Necrosis Factor-Alpha
USEPA	United States Environmental Protection Agency
WHO	World Health Organization
